# Are pro-inflammatory markers associated with psychological distress in a cross-sectional study of healthy adolescents 15–17 years of age? The Fit Futures study

**DOI:** 10.1186/s40359-022-00779-8

**Published:** 2022-03-15

**Authors:** Jonas Linkas, Luai Awad Ahmed, Gabor Csifcsak, Nina Emaus, Anne-Sofie Furberg, Guri Grimnes, Gunn Pettersen, Kamilla Rognmo, Tore Christoffersen

**Affiliations:** 1 Department of Health and Care Sciences, UiT The Arctic University of Norway, Lodve Langesgate 2, 8514 Narvik, Norway; 2 Institute of Public Health, College of Medicine and Health Sciences, United Arab Emirates University, Al Ain, UAE; 3 Department of Psychology, UiT The Arctic University of Norway, Tromsø, Norway; 4 Department of Health and Care Sciences, UiT The Arctic University of Norway, Tromsø, Norway; 5 Faculty of Health and Care Sciences, Molde University College, Molde, Norway; 6 Division of Internal Medicine, University Hospital of North Norway, Tromsø, Norway; 7 Institute of Clinical Medicine, UiT - The Arctic University of Norway, Tromsø, Norway; 8 School of Sport Sciences, UiT The Arctic University of Norway, Alta, Norway; 9 Finnmark Hospital Trust, Alta, Norway

**Keywords:** Psychological distress, Inflammatory markers, Depressive symptoms, Anxiety symptoms, Adolescence

## Abstract

**Background:**

Inflammatory markers have been associated with depression and anxiety disorder in adolescents. Less is known about the association between inflammation and subclinical symptoms in the form of psychological distress. We investigated prevalence of psychological distress and examined the associations between common pro-inflammatory markers and psychological distress in an adolescent population sample.

**Methods:**

The study was based on data from 458 girls and 473 boys aged 15–17 years from the Fit Futures Study, a large-scale study on adolescent health, conducted in Northern Norway. Psychological distress was measured with the Hopkins Symptom Checklist (HSCL-10). Serum-levels of the following low-grade inflammatory markers were measured: C-reactive protein (CRP), interleukin 6 (IL-6), transforming growth factor-alpha (TGF-α), tumor necrosis factor alpha variant 1 (TRANCE) and tumor necrosis factor alpha variant 2 (TWEAK). Associations between quartiles of inflammatory markers and HSCL-10 were examined by logistic regression and adjusted for potential confounders in sex-stratified analyses.

**Results:**

The proportion of psychological distress above cutoff were 26.9% and 10.8% among girls and boys, respectively. In both girls and boys, crude analysis showed positive associations between all inflammatory markers and HSCL-10, except for TWEAK and TRANCE in boys. However, none of these associations were statistically significant. Further, there were no significant findings in the adjusted analyses.

**Conclusion:**

There was a higher prevalence of psychological distress in girls compared to boys. Pro-inflammatory markers were not significantly associated with psychological distress in data from healthy adolescents aged 15–17 years.

**Supplementary Information:**

The online version contains supplementary material available at 10.1186/s40359-022-00779-8.

## Background

Mental disorders are a leading cause of disability globally. Many mental disorders have age-of-onset in childhood or adolescence [1] with a worldwide-pooled prevalence of 13.4% in 2015 [2]. In most countries, the median age-of-onset of having any mental disorder is during teenage years [1]. Subclinical mental burdens are even more prevalent [3]. In a recent survey among high school students in Norway, self-reported psychological distress was 31% and 12% among girls and boys respectively [4]. ‘Psychological distress’ can be defined as “a state of emotional suffering characterized by symptoms of depression and anxiety” [5]. The term refers to subclinical symptoms of anxiety and depression, but may also be an indication of mental disorder onset [5]. For some adolescents, psychological distress during growth leads to psychiatric conditions later in life [6, 7]. In order to promote good mental health among adolescents and reduce the burden of mental disorders and reduce the risk of suicidal ideation [8], more detailed investigations into the development of mental disorders in this age group are warranted.

There has been a growing interest in research on biological mechanisms that may increase our understanding of the aetiology of mental disorders and psychological distress. In the last two decades, the possible role of inflammation as a triggering factor for depression has been studied [9, 10]. Reports indicate that about one in three adults with depression have elevated levels of inflammatory cytokines [11]. Furthermore, patients with inflammatory conditions have higher risk for depressive disorders, and treatments based on cytokines may induce depressive symptoms [11]. In a meta-analysis of inflammatory markers in patients with major depressive disorder [12], it was concluded that interleukin 6 (IL-6), C-reactive protein (CRP), interleukin 1β (IL-1β) and tumor necrosis factor alpha (TNF-α) were positively associated with major depressive disorder, yet the latter two were dependent on age. In addition, the same inflammatory markers and white blood cell count have been associated with anxiety disorders in adults [13–18]. Psychological distress is widely used in population-based studies of mental health among adults [19], and has been found to be associated with CRP in large population studies [20, 21].

Studies investigating associations between inflammatory markers and depressive disorders in adolescent samples have produced conflicting results [22–27].

A meta-analysis from 2019 on children and adolescents with a diagnosis of depressive disorder included five studies and reported a trend for higher levels of TNF-α in participants with depressive disorders compared to controls [28]. Other inflammatory markers were not significantly different between healthy and diagnosed subjects [28]. A more recent meta-analysis on children and adolescents identified nine studies on inflammatory markers and anxiety-based disorders and concluded that there were no significant associations [29]. However, both meta-analyses included few studies with small sample sizes, and the authors reviewed the results as provisional and concluded that more studies are warranted [28, 29].

Regarding investigations on the association between inflammatory markers and subclinical depressive symptoms in adolescents, Mills et al. [30] conducted a systematic review with 18 studies including both subclinical depressive symptoms and clinical depression as study outcomes. The review reported that the associations between inflammatory markers and depression have many similarities with adult findings, but some noticeable differences appeared. Especially, a further broad exploration of differential roles from specific markers (e.g., IL-6, CRP and TNF-α) during growth were requested. A later cross-sectional study confirmed a positive association between high sensitive serum CRP (hs-CRP) and depression-score, controlling for anthropometric and lifestyle factors [31]. The study included solely girls with a range from 12 to 18 years of age. In contrast, a large population-based study with data from 1535 participants 13 and 16 years of age did not support an association between elevated hs-CRP and depressive symptoms [32].

One study of US adolescents has looked at the association between CRP and symptoms of generalized anxiety disorder GAD [33]. GAD includes a range of symptoms highly comorbid with depression symptoms. Indeed, increased levels of CRP were found to be associated with symptoms of GAD in bivariate cross-sectional analyses. However, all associations were attenuated when controlling for other health-related covariates, demographics, and substance use.

In summary, there is limited literature and inconsistent findings on the associations between inflammatory markers and subclinical symptoms of depression and anxiety in healthy adolescents. Furthermore, in the vast majority of descriptive reports, adolescent girls show higher levels of psychological distress than boys, and the reported associations between inflammatory markers and psychological distress seem to be dependent on sex [4, 23, 34, 35].

With the scarcity of research, and inconsistent findings on the associations between inflammatory markers and psychological distress in healthy adolescents, further research is warranted. The aims of this study were to a) describe the prevalence of psychological distress in girls and boys 15–17 years of age and b) examine the associations between inflammatory markers and psychological distress in girls and boys 15–17 years of age.

## Methods

### Study population and design

**Fig. 1:**
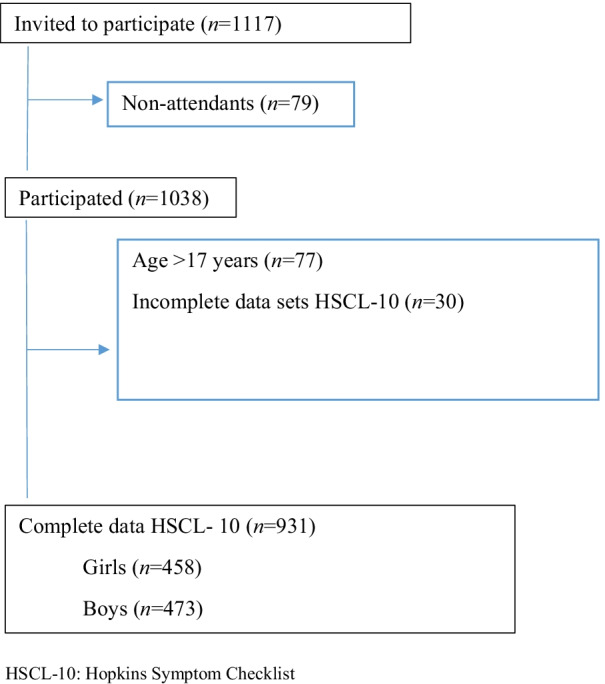
Study flowchart—Fit Futures 2010–2011

In 2010–2011, all first-year upper secondary school students in two municipalities in Northern Norway were invited to participate in a broad health study, namely the Fit Futures, an expansion of the population-based Tromsø Study. Fit Futures has previously been comprehensively described [36]. In brief, the study was conducted during school hours at the Clinical Research Unit, at the University Hospital of North Norway, Tromsø. In total, 1117 students were invited to participate, and 1038 (92.9%) attended the study (Fig. 1).

All participants provided informed consent. Participants younger than 16 years provided written informed consent from a guardian. The study was conducted in accordance with the Declaration of Helsinki and was approved by the Norwegian Data Protection Authority (reference number 2009/1282). The Regional Committee of Medical and Health Research Ethics has also approved the study (reference number 2011/1702/REK Nord), and the present project (reference number: 2019/60811/REK Nord).

Data about lifestyle, health and disease was collected with a web-based battery of questionnaires. Dedicated and trained research nurses performed clinical examinations, collected and administered blood samples, and conducted interviews on medication including use of hormonal contraceptives, and acute and chronic diseases. Height and weight were measured following standard procedures [36]. An automatic electronic scale, the Jenix DS 102 stadiometer (Dong Sahn Jenix, Seoul, Korea) was used to measure weight. Total body fat mass was measured by dual X-ray absorptiometry (DXA; GE Lunar prodigy, Lunar Corporation, Madison, WI, USA).

### Measurements and questionnaires

#### Hopkins Symptom Check List (HSCL-10)

HSCL-10 was included in the web-based questionnaire. HSCL-10 is a valid and reliable instrument [37] measuring symptoms of anxiety (4 items) and depression (6 items) during the last seven days [37]. The response categories are “none” (1), “slightly” (2), “much” (3), and “very much” (4). Cronbach’s alpha in this sample was 0.87 (for girls 0.88, for boys 0.83). To quantify psychological distress, the average score of the 10 items was calculated. A dichotomized version of HSCL-10 was created, with 1.85 as cutoff, since values at or above that threshold indicate psychological distress of clinical relevance in community samples of adolescents [37]. This cutoff has been found to have a sensitivity of 89% and a specificity of 98% when the using HSCL-25 (cutoff 1.75) as criterion [37]. There were 458 girls and 473 boys with complete data on HSCL-10.

#### Main exposure variable: pro-inflammatory markers

The participants provided non-fasting blood samples, which was collected from the antecubital vein. Serum samples were transferred to Supelco glass vials (Sigma-Aldrich Norway AS, Oslo, Norway), and stored at − 70 °C. Serum levels of inflammatory proteins were analysed by Protein Extension Array Technology (Proseek Multiplex Inflammation panel; Olink Bioscience, Uppsala, Sweden). More details about the process of analysis are described elsewhere [38]. Based on current knowledge, the following inflammatory markers were selected for analyses: CRP, IL-6, transforming growth factor-alpha (TGF-α), TNF-α variant 1 (TRANCE) and TNF-α variant 2 (TWEAK) [12, 30, 39]. The number of girls and boys respectively with data on the inflammatory markers were as following: CRP (394 and 429), IL-6 (398 and 445), TGF-α (398 and 445), TRANCE (398 and 445), and TWEAK (398 and 445).

#### Covariates

Several variables are associated with both inflammation and psychological distress and were therefore included as covariates. Demographic and anthropometric variables included were: age, age at menarche (girls), pubertal status (boys), high school program as a proxy for socioeconomic status, body fat percentage and serum vitamin D levels. Lifestyle variables included were smoking, snuffing tobacco, alcohol use, physical activity, and sleep. Health variables included were hormonal contraceptives (girls), chronic disease, current infection, and medications, use of analgesics and antibiotics that potentially can influence systemic inflammation. In addition, all analyses were sex-stratified because girls show higher levels of depressive symptoms than boys during adolescence, and there are reports that the associations between inflammatory markers and depressive symptoms are sex-dependent.

For smoking and snuffing tobacco, there were three alternative answers: “daily”, “sometimes” and “never”. Smoking and snuffing tobacco were recoded into a dichotomous variable, with “never” as the first category, while “sometimes” and “yes” were collapsed together as the second category. This was done because a low frequency of participants reported daily smoking and a low frequency reported that they snuffed sometimes. Frequency of alcohol-consumption was measured from 1 (never) to 5 (four or more times per week) and was recoded into three categories: “never”, “once per month” and “twice or more per month”.

Physical activity was measured by the Saltin-Grimby physical activity level scale [40] which addresses leisure time physical activity, asking about the type of activity and intensity in an average week during the last year. The four alternatives were: 1 (reading, watching TV, or other sedentary activity), 2 (walking, cycling or exercises at least 4 h a week), 3 (participation in recreational sports, heavy outdoor activities, snow clearing etc. at least 4 h a week) and 4 (participation in hard training or sports competitions several times each week). Physical activity was recoded into a dichotomous variable, with sedentary activities coded as zero and moderate and higher levels of activity coded as one.

For girls, pubertal status was estimated through the question: “When did you have your first menstruation”. We created a dichotomized variable, “early” (at mean 12.68 years or below) or “late” (above mean). The reliability of self-reported menarche age is established [41]. In boys, pubertal status was measured with the Pubertal Development Scale (PDS) [41, 42]. Participating boys answered four questions: growth spurt, pubic hair growth, changes in voice and facial hair growth. The four alternatives were 1 (have not begun), 2 (barely started), 3 (underway), and 4 (completed). We summarized the total score on the four items and divided by four to create a mean score. Further, we used the mean score to categorize into four categories: “not begun” (mean score below 2) “barely started” (mean score from 2 to 2.99), “underway (mean score from 3 to 3.99) and “completed” (mean score of 4). For sleep, participants reported how many whole hours they normally slept every night, with the lowest category being “four hours or less” and the highest category being “12 h or more”. The lowest category was coded as four hours, and the highest category was coded as 12 h. We created a dichotomous sleep variable divided by mean hours of sleep (6.95 h for girls and 7.09 h boys respectively). High school program consisted of three categories: “general studies”, “sports and physical” and “vocational”.

Body fat percentage was calculated as total fat mass (kg) divided by weight (kg). We created a dichotomous body fat percentage variable with cutoffs on 30 and 25% in girls and boys respectively [43]. Participants answered “yes” or “no” on questions about current infection, chronic disease and oral contraceptives, and dichotomized variables were created. Participants self-reported on their use of different types of medication. To assess for intake of medications that potentially influence systemic inflammation a dichotomized variable was created (medication intake). Vitamin D status was assessed by serum 25-hydroxyvitamin D (25-OH)D, analysed by high pressure liquid chromatography mass spectroscopy (LC–MS/MS) in stored sera (− 80 °C) at Haukeland University Hospital, Norway [44]. To standardize the results according to the Vitamin D Standardization program (VSDP), stored samples were re-analysed at the Cork Centre for Vitamin D and Nutrition Research, Ireland [45]. More details are described elsewhere [46]. The standardized version of (25-OH)D (nmol/L) was used as a continuous variable.

### Statistical analysis

We excluded participants aged 18 years or above, and with incomplete data on the outcome variable (psychological distress as measured with HSCL-10) (Fig. 1). Data was inspected for outliers and normal distribution, using QQ plots, means and trimmed means. Exposure variables and potential confounders were tested for multicollinearity. All analyses conducted were stratified by sex.

Chi-square test was used to compare the number of girls and boys scoring above the cutoff of HSCL-10. Furthermore, variables were compared between those above and below the cutoff (with- and without psychological distress). Categorical variables were compared using Chi-Square test. Continuous variables with normally distributed data were compared with independent sample *t*-tests. Continuous variables with skewness were compared with Mann–Whitney U-test.

Binary logistic regression was conducted to estimate the odds ratio (OR) and 95% confidence intervals (CIs) between pro-inflammatory markers and psychological distress. Quartiles of inflammatory marker variables were created, and the crude associations between the quartiles of inflammatory markers and psychological distress were estimated. Subsequently, adjustment for potential confounding with stepwise regressions was conducted. Potential confounders were first tested in simple logistic regressions with the categorical version of HSCL-10 as outcome, and included when the *p*-value was below 0.05. Forward selection was stopped when the added confounder had a *p*-value above 0.2 when added to the model.

Interaction was tested between the quartiles of the inflammatory markers and physical activity, body fat percentage and sleep [47–52]. In boys, there were significant interactions between CRP and sleep (*p* = 0.03) and between TGF-α and sleep (*p* = 0.04). However, these interaction terms could not be included in the adjusted analysis because of too few events (13 events in boys with psychological distress and high sleep), per covariate in the models [53].

As supplementary analyses, the mean score for the six depressive symptoms and four anxiety symptoms respectively were calculated and compared with the mean of HSCL-10. Additionally, stepwise forward logistic regressions with a dichotomous version of the six items of depressive symptoms as outcome were conducted. Also, the same stepwise regressions with continuous inflammatory markers as exposure for both the respective outcomes were conducted. Lastly, linear regressions with mean HSCL-10 as outcome were conducted.

A significance level of *p* < 0.05 as an indication of statistical significance was chosen. All statistical analyses were conducted with the Statistical Package of Social Science (SPSS v. 26).

## Results

### Prevalence of psychological distress in girls and boys

**Fig. 2:**
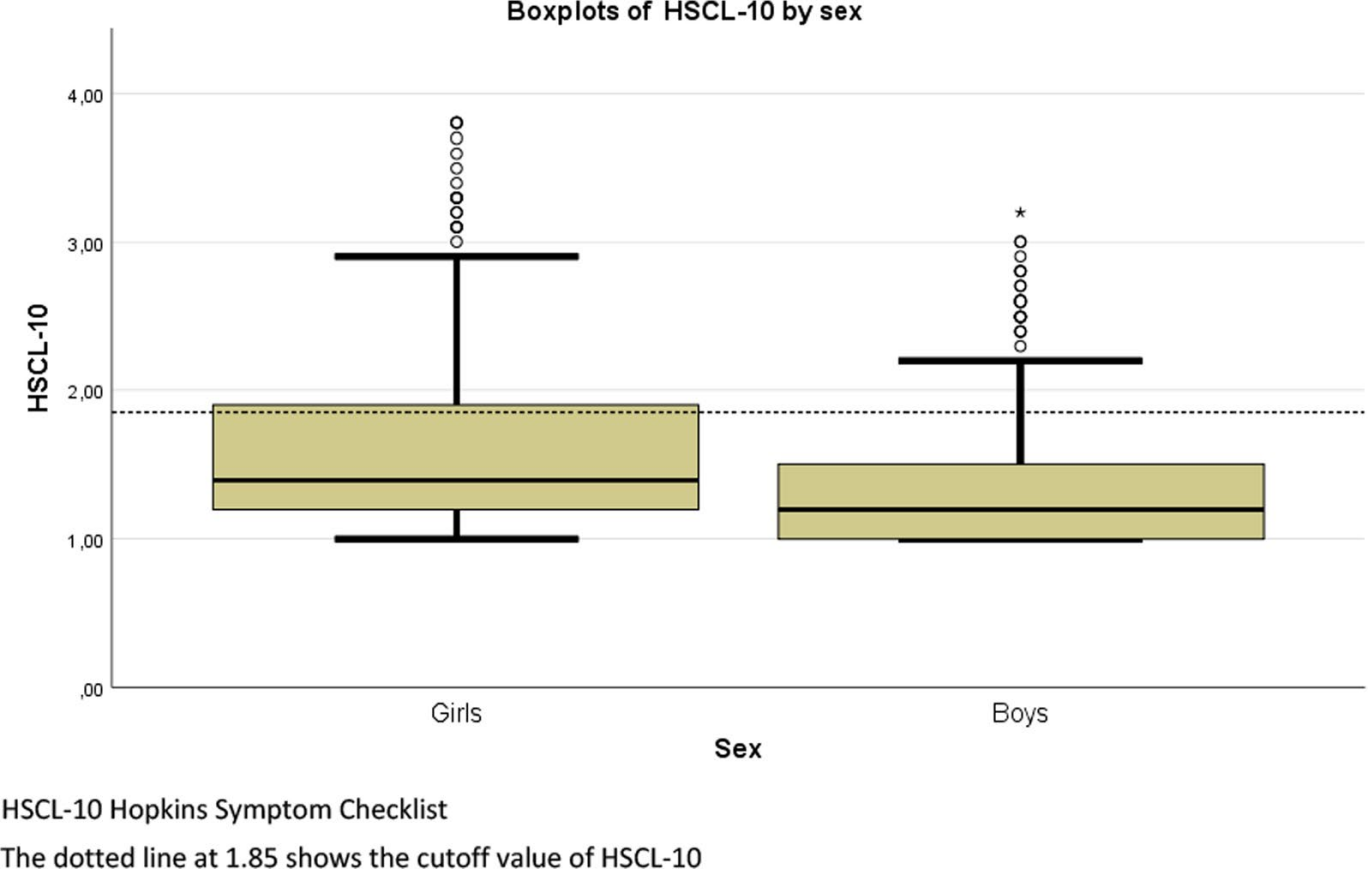
Median and IQR of HSCL-10, for girls and boys respectively. Fit Futures 2010–2011

Girls reported a mean HSCL-10 (SD) of 1.63 (0.59), and boys reported 1.35 (0.41). Corresponding median (IQR) in girls and boys were 1.40 (0.70), and 1.20 (2.20), respectively (Fig. 2). In girls, 26.9% (*n* = 123) scored above the 1.85 cutoff value of HSCL-10. In boys, 10.8% (*n* = 51) scored above the cutoff value. The prevalence of psychological distress was significantly different between girls and boys, *χ*^*2*^ (1, *N* = 931) = 39.6, *p* < 0.01.

Characteristics by psychological distress for girls and boys are shown in Tables 1 and 2 respectively.

**Table 1:** Characteristics by psychological distress for girls

	With psychological distress		Without psychological distress		*p*-value*
	*n*	Mean (SD)	*n*	Mean (SD)	
Age in years mean (SD)	123	16.15 (0.44)	335	16.13 (0.41)	0.23
Body fat percentage, dichotomous	123		332		0.03
< 30		33.3%		44.9%	
≥ 30		66.7%		55.1%	
Age menarche (years)	121		331		0.88
Early (≤ 12.68)		39.7%		40.5%	
Late (> 12.68)		60.3%		59.5%	
Smoking	123		334		< 0.01
No, never		62.6%		85.9%	
Yes		37.4%		14.1%	
Snuffing tobacco	123		334		< 0.01
No, never		53.7%		71.3%	
Yes		46.3%		28.7%	
Alcohol	123		335		0.01
Never		14.6%		26.6%	
Once per month or less		46.3%		46.6%	
Twice or more per month		39.0%		26.9%	
Physical activity	123		335		< 0.01
Sedentary		22.0%		11.3%	
Active		78.0%		88.7%	
Sleep (h)	123		334		< 0.01
Low (< 7)		51.2%		34.7%	
High (> 7)		48.8%		65.3%	
Current infection	122		334		0.20
No		82.8%		87.4%	
Yes		17.2%		12.6%	
Chronic disease	122		334		0.01
No		59.9%		72.2%	
Yes		41.0%		27.8%	
Hormonal contraceptives	122		333		< 0.01
No		50.8%		65.8%	
Yes		49.2%		34.2%	
Intake of medication	122		334		0.05
No		59.8%		69.8%	
Yes		40.2%		30.2%	
High school program	123		335		0.02
General studies		45.5%		54.3%	
Sports and physical		4.9%		9.6%	
Vocational		49.6%		36.1%	
CRP mg/ L median and IQR	97	0.72 (1.39)	297	0.47 (1.08)	0.11
IL-6 pg/L median and IQR	98	2.79 (0.59)	300	2.68 (0.57)	0.05
TGF-α pg/L median and IQR	98	3.90 (0.80)	300	3.90 (0.71)	0.91
TRANCE pg/L median and IQR	98	5.62 (0.77)	300	5.53 (0.76)	1.00
TWEAK pg/L median and IQR	98	8.94 (0.51)	300	8.88 (0.42)	0.87
Vitamin D nmol/L median and IQR	98	39.36 (25.94)	300	41.90 (24.07)	0.65

Fit Futures 2010–2011 (n = 458)

^*^Chi-square for categorical variables and t-test or Mann Whitney U for continuous variables

With psychological distress: a score above 1.85 on HSCL-10

Without psychological distress: a score below 1.85 on HSCL-10

Mean (SD) of continuous variable and percentages of categorical variables are reported

Median and IQR are reported for inflammatory markers and Vitamin D

Intake of medication: Intake of medications, analgetics or antibiotics in the last 24 h

*CRP* C-reactive protein, *IL6-α* Interleukin 6 alpha, *TGF-α* Transforming growth factor alpha, *TRANCE* Tumor Necrosis Factor-related activation-induced cytokine (O14788: TNF-related activation-induced cytokine within limits of detection), *TWEAK* Tumor necrosis factor-like weak inducer of apoptosis (O43508: TNF-like weak inducer of apoptosis within limits of detection), *Vitamin D* Standardized version of (25-OH)D

**Table 2:** Characteristics by psychological distress for boys

	With psychological distress		Without psychological distress		*p*-value*
	*n*	Mean (SD)	*n*	Mean (SD)	
Age in years Mean (SD)	51	16.14 (0.49)	422	16.05 (0.45)	0.09
Body fat percentage, dichotomous	50		422		0.41
< 25		68%		73.5%	
≥ 25		32%		26.5%	
PDS status	42		334		0.52
Completed		23.8%		16.8%	
Underway		69.0%		74.6%	
Barely started		7.1%		8.7%	
Not begun		0%		0%	
Smoking	51		422		0.07
No, never		66.7%		78.0%	
Yes		33.3%		22.0%	
Snuffing tobacco	51		421		0.50
No, never		54.9%		59.9%	
Yes		45.1%		40.1%	
Alcohol	51		420		0.32
Never		31.4%		32.9%	
Once per month or less		29.4%		37.6%	
Twice or more per month		39.2%		29.5%	
Physical activity	51		422		< 0.01
Sedentary		49.0%		27.0%	
Active		51.0%		73.0%	
Sleep (h)	51		415		0.01
Low (≤ 7)		74.5%		54.9%	
High (> 7)		25.5%		45.1%	
Current infection	51		421		0.86
No		86.3%		87.2%	
Yes		13.7%		12.8%	
Chronic disease	51		420		0.02
No		58.8%		74.8%	
Yes		41.2%		25.2%	
Intake of medication	51		421		0.57
No		78.4%		81.7%	
Yes		21.6%		18.3%	
High school program	51		422		0.12
General studies		39.2%		28.7%	
Sports and physical		5.9%		14.7%	
Vocational		54.9%		56.6%	
CRP mg/ L Median and IQR	44	0.47 (1.22)	385	0.49 (0.78)	0.64
IL-6 pg/L Median and IQR	47	2.76 (0.61)	398	2.72 (0.62)	0.82
TGF-α pg/L Median and IQR	47	3.75 (0.68)	398	3.61 (0.74)	0.09
TRANCE pg/L median and IQR	47	6.01 (0.77)	398	6.04 (0.65)	0.99
TWEAK pg/L median and IQR	47	9.00 (0.30)	398	9.02 (0.37)	0.15
Vitamin D nmol/L median and IQR	47	25.91 (15.81)	399	30.44 (21.43)	0.01

Fit Futures 2010–2011, (n = 473)

^*^Chi-square for categorical variables and *t*-test or Mann Whitney U for continuous variables

With psychological distress: a score above 1.85 on HSCL-10

Without psychological distress: a score below 1.85 on HSCL-10

Mean (SD) of continuous variable and percentages of categorical variables are reported

Median and IQR are reported for inflammatory markers and Vitamin D

*PDS status* Pubertal Development Scale status, *Intake of medication* Intake of medications, analgetics or antibiotics in the last 24 h, *CRP* C-reactive protein, *IL6-α* Interleukin 6 alpha, *TGF-α* Transforming growth factor alpha, *TRANCE* Tumor Necrosis Factor-related activation-induced cytokine (O14788: TNF-related activation-induced cytokine within limits of detection), *TWEAK* Tumor necrosis factor-like weak inducer of apoptosis (O43508: TNF-like weak inducer of apoptosis within limits of detection), *Vitamin D* Standardized version of (25-OH)D

In girls with psychological distress, a higher percentage scored above the cutoff for body fat percentage, slept below 7 h, smoked, snuffed tobacco, drank alcohol, were less physically active, had chronic diseases, used oral contraceptives and used medication compared to girls without psychological distress. The distribution across high school programs was significantly different for girls with- and without psychological distress (Table 1).

In boys with psychological distress, a higher percentage slept below 7 h, were less physically active and had chronic diseases compared to boys without psychological distress.

### Associations between inflammatory markers and psychological distress

In girls, crude analyses showed positive associations between all inflammatory markers (in quartiles), and HSCL-10. The highest OR (95% CI) was found for IL-6, 1.21 (0.98, 1.48). In boys, all crude associations were positive, except for those between TWEAK, TRANCE and HSCL-10. The highest OR (95% CI) was found for TGF-α, 1.20 (0.91, 1.58). However, none of these associations were statistically significant. Further, there were no significant findings in the adjusted analyses (Table 3). Body fat percentage did not confound the associations in any of the adjusted analyses.

**Table 3:** Crude and adjusted associations between quartiles of inflammatory proteins and HSCL-10

	Crude analysis			Adjusted analysis		
	*n*	Odds ratio (95% CI)	*p*-value	*n*	Odds ratio (95% CI)	*p*-value
*Girls*						
CRP quartiles	394	1.18 (0.96, 1.45)	0.11	389	1.11 (0.90, 1.39)	0.33
IL-6 quartiles	398	1.21 (0.98, 1.48)	0.08	393	1.15 (0.92, 1.42)	0.22
TGF-α quartiles	398	1.01 (0.82, 1.24)	0.92	393	1.03 (0.83, 1.28)	0.80
TRANCE quartiles	398	1.07 (0.87, 1.31)	0.53	393	1.13 (0.90, 1.40)	0.29
TWEAK quartiles	398	1.02 (0.83, 1.25)	0.84	393	1.11 (0.89, 1.38)	0.35
*Boys*						
CRP quartiles	429	1.04 ( 0.79, 1.37)	0.79	420	1.00 (0.75, 1.33)	0.98
IL-6 quartiles	444	1.05 (0.80, 1.38)	0.73	435	0.99 (0.75, 1.32)	0.95
TGF-α quartiles	444	1.20 (0.91, 1.58)	0.19	435	1.190 (0.89, 1.59)	0.23
TRANCE quartiles	445	0.99 (0.76, 1.30)	0.95	436	0.99 (0.75, 1.30)	0.91
TWEAK quartiles	445	0.87 (0.66, 1.14)	0.31	436	0.88 (0.62, 1.16)	0.35

For girls, the adjusted models for CRP, IL-6, TGF-α and TRANCE included the following covariates: smoking, physical activity and chronic disease

The adjusted TWEAK model included the following covariates: smoking, snuffing tobacco, physical activity and chronic disease

For boys, adjusted model for all inflammatory markers included the following covariates: physical activity, sleep and chronic disease

### Supplementary analysis

Using the six items measuring depressive symptoms as outcome did not alter the non-significant associations. All p-values were above 0.06 (Additional file 1). Neither using continuous inflammatory markers as exposure did alter the non-significant outcomes. All p-values were above 0.14 with HSCL-10 as outcome (Additional file 2) and all p-values were above 0.10 with the six depressive symptoms items as outcome (Additional file 3). Lastly, there were no significant crude nor adjusted associations in the linear regressions with mean HSCL-10 as outcome. All p-values were above 0.14 (Additional file 4).

## Discussion

In this sample of 15–17 years old adolescents, girls reported a statistically significantly higher prevalence of psychological distress than boys. There were no statistically significant associations between any of the pro-inflammatory markers and psychological distress, neither for girls nor boys. After adjustment for potential confounders, the associations remained statistically non-significant.

### Prevalence of psychological distress

The sex difference in prevalence of psychological distress is consistent with previous studies on this age group [32, 54]. Kleppang et al. found a corresponding sex difference in a Norwegian sample of 15–16 year olds, measuring psychological distress with HSCL-10 in 2009 [34]. Similar sex differences were also found in a study when a subset of HSCL-10 was used (the six items that measure depressive symptoms were included, whilst anxiety items were excluded). This was a study from 2015 on a Norwegian sample aged 13–16 years [55]. The proportions of psychological distress in girls and boys in this age group seems to be consistent [56, 57].

The finding that psychological distress differs according to several lifestyle factors is consistent with another study on Norwegian adolescents aged 13–18 years [58]. Differences in psychological distress according to socioeconomic status (high school as a proxy in the present study) [59], body fat percentage [60] oral contraceptives use [61], medication use [62–65], and the prevalence of chronic diseases [66] are also reported in other studies.

### Associations between pro-inflammatory markers and psychological distress

This study investigated five inflammatory markers, separately for girls and boys, and could not show any significant associations with psychological distress in crude or adjusted analyses. Thus, this study does not provide any indications for cross-sectional associations between inflammatory markers and psychological distress in adolescents.

In line with the results in this study, there are several studies that report no associations between inflammatory markers and depressive symptoms. Chaiton et al. [32] found no crude or adjusted associations between CRP and depressive symptoms in a sample of 1532 healthy adolescents aged 13–16. They used the Center for Epidemiologic Studies Depression Scale (CES-D) to measure depressive symptoms. CES-D consists of 20 items measuring depressive symptoms in the general population. This outcome is somewhat different from HSCL-10, which measures psychological distress more generally, including 4 items about anxiety symptoms. However, since most of the HSCL-10 items measure depressive symptoms, and both scales measure primarily subclinical burdens and can be used to identify individuals with risk of clinical burdens, they probably have a high degree of concordance. Supporting this, measures of anxiety and depression generally have high correlations during adolescence [67]. In the supplementary analyses, conducting the regressions with the 6 depressive symptoms as outcome did not alter the findings, indicating that including anxiety items to the outcome variable was not the reason why we did not find any significant associations. Correspondingly, another study found no cross-sectional associations between IL-6 and Children’s Depression Inventory (CDI), as a measure of depressive symptoms [68]. As in the present study, this study was also conducted in a community sample, with 288 participants, 51.4% girls and a mean age of 16.33 years. In sum, across samples of different ages, and with the use of different measures of psychological distress, there is generally a lack of associations in healthy adolescents. This suggests that the null-findings are neither related to age nor to the use of HSCL-10 as a measure of psychological distress.

In contrast to the present null findings, there are studies that found associations between inflammatory markers and depressive symptoms in adolescents. Tabatabaeizadeh et al. [31] found an association between CRP and depressive symptoms in 563 girls aged 12–18 years. This was a cross-sectional case–control study, which used the Beck depression Inventory-II (BDI-II). The study included 244 cases with mild to severe depression, and 319 age matched controls without depressive symptoms. Severe depressive symptoms had the strongest association with CRP, followed by moderate and mild symptoms. This is in line with previous research showing that the strongest associations with inflammatory markers are found with more severe symptoms and in clinical samples [69]. In contrast, the sample in the present study had low levels of psychological distress and is therefore not directly comparable with case-studies with higher proportions of participants having depressive symptoms.

### Interpretations

When using cross-sectional data, the duration of the elevation of inflammatory markers is unknown. It is possible that inflammation needs to persist over a certain time period to influence the brain enough to result in increased levels of depressive symptoms [39, 70–72]. Indeed, it has been suggested that the strongest associations between inflammatory markers and depressive symptoms in adolescents are found in prospective studies where time to follow-up is at least 13 months [35]. Findings indicate that the same mechanism is present at older age (aged > 60 years), with corresponding findings of prospective associations and lack of cross-sectional associations [73]. Further, the elevated risk for depressive disorder found in patients with inflammatory conditions [11] supports that enduring inflammation is associated with increased levels of depressive symptoms. It is possible that the duration of elevated inflammatory markers may have been short, or even acute, for many of the participants in the present study.

Another possibility mentioned by Chaiton et al. [32] is that the pathophysiology of depression is different in adolescents and adults. Therefore, the association between inflammation and depressive symptoms found in healthy adults will not necessarily be present among healthy adolescents. The association may also be weaker during adolescence since adolescents in general have lower levels of inflammation compared to adults [30]. Lower levels of inflammation make it more difficult to detect associations with depressive symptoms in adolescents. Furthermore, it is possible that the positive associations we found in this study might have been significant with a larger sample size.

The lack of associations may be caused by using HSCL-10, which is a combination of depressive and anxiety symptoms, as outcome. Depression consists of different symptoms, which seem to associate differently with inflammation [74, 75]. For example, inflammation has been found to associate specifically with sleeping problems and lack of energy [76]. The trend in psychoneuroimmunology research is therefore to investigate associations with different types of symptoms. Investigating different symptoms also increases power compared to using total symptoms [77].

#### Strengths and limitations

This study has several strengths. Firstly, the study was conducted on a sample of healthy adolescents and the attendance rate was very high. Adolescents represent an understudied population in general and with respect to the association between inflammation and depressive symptoms literature is scarce. One advantage of studying this age group is less noise from confounders, such as chronic diseases and obesity, typically more common in older subjects [30]. Secondly, all analyses were sex-stratified, since earlier studies have shown sex-differences in prevalence of both psychological distress and associations between inflammatory markers and depressive symptoms. Finally, we included an array of inflammatory markers that have been recommended by previous systematic reviews [30], that allowed us to explore the associations of different inflammatory markers with psychological distress.

This study has some limitations. The cross-sectional design restrict interpretations and we cannot infer causality. Further, the design measures outcome and exposure at the same time and cannot assess persistent inflammation. Secondly, we used self-report of psychological distress. This may not be as clinically relevant as diagnostic interviews. However, HSCL-10 is a reliable and valid instrument to measure psychological distress, with high sensitivity and specificity with the applied cutoff of 1.85 [37]. Thirdly, the sample size of this study may be too small to detect weak associations as previously found in large adult population samples, with approximately 70,000 participants [20, 21]. Even though larger sample sizes permit the detection of statistically significant associations between inflammatory markers and depressive symptoms, these associations may not be clinically relevant. Thus, smaller sample sizes, like the one used in this study, are still useful to investigate potential associations of clinical significance. Fourthly, we used dichotomous variables for current infection and chronic disease respectively. Thus, when assessing potential confounding, we did not examine different types of infection and chronic disease. Future studies, with bigger samples may benefit from discriminating between different kinds of infections and chronic diseases. However, the relatively few cases with psychological distress in our study (especially in boys) justify our adjustment for dichotomous versions of infection and chronic disease. Finally, since blood samples used in this study were collected from non-fasting participants, they may have been affected by diurnal effects [78]. Nonetheless, measurement errors are probably random in association with the outcome [79].

## Conclusion

According to this study, the prevalence of psychological distress is higher in girls than in boys aged 15–17 years of age. The prevalence found in both girls and boys corroborates with previous findings in this age group. No evidence was found for associations between pro-inflammatory markers and psychological distress in healthy adolescents aged 15–17 years.

It is recommended to conduct prospective studies to elucidate possible longitudinal mechanisms and directionality. Future studies should also consider using larger sample sizes to detect possible significant positive associations.

## Supplementary Information

**Additional file 1.** Associations between quartiles of inflammatory-proteins and six depression-items from HSCL-10, by logistic forward stepwise regression.

**Additional file 2.** Crude and adjusted associations between continuous inflammatory-proteins and HSCL-10, by logistic forward stepwise regression.

**Additional file 3.** Associations between continuous inflammatory-proteins and six depression-items from HSCL-10, by logistic forward stepwise regression.

**Additional file 4.** Crude and adjusted associations quartiles of inflammatory proteins and HSCL-10, by linear regressions.

## Data Availability

The dataset supporting the conclusions of this article is available in the Tromsø-study repository, https://uit.no/research/tromsostudy.

## Abbreviations

(25-OH)D: Serum 25-hydroxyvitamin D
BDI-II: Beck depression Inventory-II
CDI: Children’s Depression Inventory
CES-D: Center for Epidemiologic Studies Depression Scale
CRP: C-reactive protein (CRP)
GAD: Generalized anxiety disorder
HSCL-10: Hopkins Symptom Checklist
IL-6: Interleukin 6 (IL-6)
PDS: Pubertal Development Scale
TGF-α: Transforming growth factor-alpha
TNF-α: Tumor necrosis factor alpha
TRANCE: Tumor necrosis factor alpha variant 1
TWEAK: Tumor necrosis factor alpha variant 2
VSDP: Vitamin D Standardization program
