# Correction to: Are pro-infammatory markers associated with psychological distress in a cross-sectional study of healthy adolescents 15–17 years of age? The Fit Futures study

**DOI:** 10.1186/s40359-022-00812-w

**Published:** 2022-04-20

**Authors:** Jonas Linkas, Luai Awad Ahmed, Gabor Csifcsak, Nina Emaus, Anne-Sofe Furberg, Guri Grimnes, Gunn Pettersen, Kamilla Rognmo, Tore Christofersen

**Affiliations:** 1 Department of Health and Care Sciences, UiT The Arctic University of Norway, Lodve Langesgate 2, 8514 Narvik, Norway; 2 Institute of Public Health, College of Medicine and Health Sciences, United Arab Emirates University, Al Ain, UAE; 3 Department of Psychology, UiT The Arctic University of Norway, Tromsø, Norway; 4 Department of Health and Care Sciences, UiT The Arctic University of Norway, Tromsø, Norway; 5 Faculty of Health and Care Sciences, Molde University College, Molde, Norway; 6 Division of Internal Medicine, University Hospital of North Norway, Tromsø, Norway; 7 Institute of Clinical Medicine, UiT - The Arctic University of Norway, Tromsø, Norway; 8 School of Sport Sciences, UiT The Arctic University of Norway, Alta, Norway; 9 Finnmark Hospital Trust, Alta, Norway

## Correction to: BMC Psychology (2022) 10:65 10.1186/s40359-022-00779-8

Following publication of the original article [1], the authors flagged that the article had published with errors in Tables 1 and 2: IL6-α TGF-α TRANCE TWEAK had been given the unit pg/L rather than the (correct) unit NPX.

The tables have now been corrected in the published article, and the corrected tables may be seen in this erratum for reference. The authors thank you for reading this correction and apologize for any inconvenience caused.

**Table 1:** Characteristics by psychological distress for girls. Fit Futures 2010–2011 (n = 458)

	With psychological distress		Without psychological distress		*p*-value*
	*n*	Mean (SD)	*n*	Mean (SD)	
Age in years mean (SD)	123	16.15 (0.44)	335	16.13 (0.41)	0.23
Body fat percentage, dichotomous	123		332		0.03
< 30		33.3%		44.9%	
≥ 30		66.7%		55.1%	
Age menarche (years)	121		331		0.88
Early (≤ 12.68)		39.7%		40.5%	
Late (> 12.68)		60.3%		59.5%	
Smoking	123		334		< 0.01
No, never		62.6%		85.9%	
Yes		37.4%		14.1%	
Snuffing	123		334		< 0.01
No, never		53.7%		71.3%	
Yes		46.3%		28.7%	
Alcohol	123		335		0.01
Never		14.6%		26.6%	
Once per month or less		46.3%		46.6%	
Twice or more per month		39.0%		26.9%	
Physical activity	123		335		< 0.01
Sedentary		22.0%		11.3%	
Active		78.0%		88.7%	
Sleep (hours)	123		334		< 0.01
Low (≤ 7)		51.2%		34.7%	
High (> 7)		48.8%		65.3%	
Current infection	122		334		0.20
No		82.8%		87.4%	
Yes		17.2%		12.6%	
Chronic disease	122		334		0.01
No		59.9%		72.2%	
Yes		41.0%		27.8%	
Hormonal contraceptives	122		333		< 0.01
No		50.8%		65.8%	
Yes		49.2%		34.2%	
Intake of medication	122		334		0.05
No		59.8%		69.8%	
Yes		40.2%		30.2%	
High School Program	123		335		0.02
General studies		45.5%		54.3%	
Sports and physical		4.9%		9.6%	
Vocational		49.6%		36.1%	
CRP mg/L median and IQR	97	0.72 (1.39)	297	0.47 (1.08)	0.11
IL-6 NPX median and IQR	98	2.79 (0.59)	300	2.68 (0.57)	0.05
TGF- α NPX median and IQR	98	3.90 (0.80)	300	3.90 (0.71)	0.91
TRANCE NPX median and IQR	98	5.62 (0.77)	300	5.53 (0.76)	1.00
TWEAK NPX median and IQR	98	8.94 (0.51)	300	8.88 (0.42)	0.87
Vitamin D nmol/L median and IQR	98	39.36 (25.94)	300	41.90 (24.07)	0.65

^*^Chi-square for categorical variables and *t*-test or Mann Whitney U for continuous variables

With psychological distress: a score above 1.85 on HSCL-10

Without psychological distress: a score below 1.85 on HSCL-10

Mean (SD) of continuous variable and percentages of categorical variables are reported

Median and IQR are reported for inflammatory markers and Vitamin D

Intake of medication: Intake of medications, analgetics or antibiotics in the last 24 h

*CRP* C-reactive protein, *IL6-α* Interleukin 6 alpha, *TGF-α* Transforming growth factor alpha, *TRANCE* Tumor Necrosis Factor-related activation-induced cytokine (O14788: TNF-related activation-induced cytokine within limits of detection), *TWEAK* Tumor necrosis factor-like weak inducer of apoptosis (O43508: TNF-like weak inducer of apoptosis within limits of detection), *NPX* Normalized protein expression, *Vitamin D* Standardized version of (25-OH)D

**Table 2:** Characteristics by psychological distress for boys. Fit Futures 2010–2011, (n = 473)

	With psychological distress		Without psychological distress		*p*-value*
	*n*	Mean (SD)	*n*	Mean (SD)	
Age in years mean (SD)	51	16.14 (0.49)	422	16.05 (0.45)	0.09
Body fat percentage, dichotomous	50		422		0.41
< 25		68%		73.5%	
≥ 25		32%		26.5%	
PDS status	42		334		0.52
Completed		23.8%		16.8%	
Underway		69.0%		74.6%	
Barely started		7.1%		8.7%	
Not begun		0%		0%	
Smoking	51		422		0.07
No, never		66.7%		78.0%	
Yes		33.3%		22.0%	
Snuffing	51		421		0.50
No, never		54.9%		59.9%	
Yes		45.1%		40.1%	
Alcohol	51		420		0.32
Never		31.4%		32.9%	
Once per month or less		29.4%		37.6%	
Twice or more per month		39.2%		29.5%	
Physical activity	51		422		< 0.01
Sedentary		49.0%		27.0%	
Active		51.0%		73.0%	
Sleep (h)	51		415		0.01
Low (≤ 7)		74.5%		54.9%	
High (> 7)		25.5%		45.1%	
Current infection	51		421		0.86
No		86.3%		87.2%	
Yes		13.7%		12.8%	
Chronic disease	51		420		0.02
No		58.8%		74.8%	
Yes		41.2%		25.2%	
Intake of medication	51		421		0.57
No		78.4%		81.7%	
Yes		21.6%		18.3%	
High school program	51		422		0.12
General studies		39.2%		28.7%	
Sports and physical		5.9%		14.7%	
Vocational		54.9%		56.6%	
CRP mg/L median and IQR	44	0.47 (1.22)	385	0.49 (0.78)	0.64
IL-6 NPX median and IQR	47	2.76 (0.61)	398	2.72 (0.62)	0.82
TGF-α NPX median and IQR	47	3.75 (0.68)	398	3.61 (0.74)	0.09
TRANCE NPX median and IQR	47	6.01 (0.77)	398	6.04 (0.65)	0.99
TWEAK NPX median and IQR	47	9.00 (0.30)	398	9.02 (0.37)	0.15
Vitamin D nmol/L Median and IQR	47	25.91 (15.81)	399	30.44 (21.43)	0.01

^*^Chi-square for categorical variables and *t*-test or Mann Whitney U for continuous variables

With psychological distress: a score above 1.85 on HSCL-10

Without psychological distress: a score below 1.85 on HSCL-10

Mean (SD) of continuous variable and percentages of categorical variables are reported

Median and IQR are reported for inflammatory markers and Vitamin D

*PDS status* Pubertal Development Scale status, *Intake of medication* intake of medications, analgetics or antibiotics in the last 24 h, *CRP* C-reactive protein, *IL6-α* Interleukin 6 alpha, *TGF-α* Transforming growth factor alpha, *TRANCE* Tumor Necrosis Factor-related activation-induced cytokine (O14788: TNF-related activation-induced cytokine within limits of detection), *TWEAK* Tumor necrosis factor-like weak inducer of apoptosis (O43508: TNF-like weak inducer of apoptosis within limits of detection), *NPX* Normalized protein expression, *Vitamin D* Standardized version of (25-OH)D
