# Supplementary material for: Are pro-inflammatory markers associated with psychological distress in a cross-sectional study of healthy adolescents 15–17 years of age? The Fit Futures study
Source: BMC Psychol. 2022 Mar 15;10:65. doi: 10.1186/s40359-022-00779-8 (PMC8925220; doi:10.1186/s40359-022-00779-8)
Supplement: Supplementary file 1 — Additional file 1. Associations between quartiles of inflammatory-proteins and six depression-items from HSCL-10, by logistic forward stepwise regression. [file 40359_2022_779_MOESM1_ESM.docx]

**Additionl file 1:** *Associations between quartiles of inflammatory-proteins and six depression-items from HSCL-10, by logistic forward stepwise regression.*

|  | Crude analysis | | | Adjusted analysis | | |
| --- | --- | --- | --- | --- | --- | --- |
|  | Girls |  |  |  |  |  |
|  | *n* | Odds ratio (95 % CI) | *p*-value | *n* | Odds ratio (95 % CI) | *p*-value |
| CRP quartiles | 394 | 1.07 (0.88, 1.31) | 0.50 | 391 | 1.00 (0.81, 1.24) | 0.99 |
| IL-6 quartiles | 398 | 1.22 (0.99, 1.50) | 0.06 | 395 | 1.17 (0.95, 1.45) | 0.14 |
| TGF-α quartiles | 398 | 1.02, (0.83, 1.24) | 0.88 | 395 | 1.01 (0.82, 1.25) | 0.93 |
| TRANCE quartiles | 398 | 1.11 (0.90, 1.36) | 0.33 | 395 | 1.17 (0.94, 1.45) | 0.15 |
| TWEAK quartiles | 398 | 0.98 (0.80, 1.21) | 0.88 | 395 | 1.02 (0.82, 1.26) | 0.87 |
|  | Boys |  |  |  |  |  |
|  | *n* | Odds ratio (95% CI) | *p*-value | *n* | Odds ratio (95 % CI) | *p*-value |
| CRP quartiles | 429 | 1.11 (0.88, 1.41) | 0.38 | 418 | 1.06 (0.83, 1.36) | 0.64 |
| IL-6 quartiles | 444 | 1.19 (0.94, 1.50) | 0.15 | 435 | 1.15 (0.90, 1.48) | 0.26 |
| TGF-α quartiles | 444 | 1,19 (0.94, 1.50) | 0.15 | 435 | 1.16 (0.91, 1.49) | 0.24 |
| TRANCE quartiles | 445 | 0.92 (0.73, 1.16) | 0.48 | 436 | 0.91 (0.71, 1.16) | 0.44 |
| TWEAK quartiles | 445 | 0.84 (0.67, 1.07) | 0.16 | 436 | 0.86 (0.67, 1.09) | 0.21 |

For girls, adjusted models for CRP and TRANCE included the following covariates: smoking, physical activity and chronic disease

Adjusted model for IL-6 included the following covariates: smoking and physical activity

Adjusted models for TGF-α and TWEAK: smoking, physical activity and sleep

For boys, adjusted model for CRP included the following covariates: smoking, physical activity and sleep

Adjusted model for IL-6, TGF-α, TRANCE and TWEAK included the following covariates: smoking, physical activity, sleep and chronic disease.
