# Supplementary material for: Are pro-inflammatory markers associated with psychological distress in a cross-sectional study of healthy adolescents 15–17 years of age? The Fit Futures study
Source: BMC Psychol. 2022 Mar 15;10:65. doi: 10.1186/s40359-022-00779-8 (PMC8925220; doi:10.1186/s40359-022-00779-8)
Supplement: Supplementary file 2 — Additional file 2. Crude and adjusted associations between continuous inflammatory-proteins and HSCL-10, by logistic forward stepwise regression. [file 40359_2022_779_MOESM2_ESM.docx]

**Additional file 2:** *Crude and adjusted* *associations between continuous inflammatory-proteins and HSCL-10, by logistic forward stepwise regression.*

|  | Crude analysis | | | Adjusted analysis | | |
| --- | --- | --- | --- | --- | --- | --- |
|  | Girls |  |  |  |  |  |
|  | *n* | Odds ratio (95 % CI) | *p*-value | *n* | Odds ratio (95 % CI) | *p*-value |
| CRP | 394 | 1.04 (0.97, 1.13) | 0.29 | 389 | 1.03 (0.95, 1.12) | 0.52 |
| IL-6 | 398 | 1.25 (0.88, 1.77) | 0.22 | 393 | 1.06 (0.73, 1.54) | 0.75 |
| TGF-α | 398 | 1.04 (0.70, 1.53) | 0.86 | 393 | 1.02 (0.67, 1.55) | 0.926 |
| TRANCE | 398 | 0.89 (0.62, 1.30) | 0.56 | 393 | 0.97 (0.66, 1.44) | 0.89 |
| TWEAK | 398 | 0.94 (0.47, 1.87) | 0.85 | 393 | 1.22 (0.59, 2.53) | 0.59 |
|  | Boys |  |  |  |  |  |
|  | *n* | Odds ratio (95% CI) | *p*-value | *n* | Odds ratio (95 % CI) | *p*-value |
| CRP | 429 | 0.99 (0.89, 1.09) | 0.77 | 420 | 0.97 (0.87, 1.08) | 0.59 |
| IL-6 | 445 | 0.81 (0.46, 1.42) | 0.47 | 436 | 0.73 (0.39, 1.39) | 0.34 |
| TGF-α | 445 | 1.45 (0.88, 2.40) | 0.15 | 436 | 1.40 (0.83, 2.35) | 0.21 |
| TRANCE | 445 | 0.94 (0.53, 1.67) | 0.84 | 436 | 0.95 (0.54, 1.69) | 0.87 |
| TWEAK | 445 | 0.47 (0.17, 1.30) | 0.14 | 436 | 0.48 (0.17, 1.36) | 0.17 |

For girls, all adjusted models included the following covariates: smoking, physical activity and chronic disease.

For boys, all adjusted models included the following covariates: physical activity, sleep and chronic disease
