# Supplementary material for: Are pro-inflammatory markers associated with psychological distress in a cross-sectional study of healthy adolescents 15–17 years of age? The Fit Futures study
Source: BMC Psychol. 2022 Mar 15;10:65. doi: 10.1186/s40359-022-00779-8 (PMC8925220; doi:10.1186/s40359-022-00779-8)
Supplement: Supplementary file 3 — Additional file 3. Associations between continuous inflammatory-proteins and six depression-items from HSCL-10, by logistic forward stepwise regression. [file 40359_2022_779_MOESM3_ESM.docx]

**Additional file 3:** *Associations between continuous inflammatory-proteins and six depression-items from HSCL-10, by logistic forward stepwise regression.*

|  | Crude analysis | | | Adjusted analysis | | |
| --- | --- | --- | --- | --- | --- | --- |
|  | Girls |  |  |  |  |  |
|  | *n* | Odds ratio (95 % CI) | *p*-value | *n* | Odds ratio (95 % CI) | *p*-value |
| CRP | 394 | 1.02 (0.94, 1.10) | 0.62 | 391 | 1.00 (0.92, 1.09) | 0.93 |
| IL-6 | 398 | 1.28 (0.90, 1.81) | 0.17 | 395 | 1.09 (0.76, 1.58) | 0.63 |
| TGF-α | 398 | 1.06 (0.72, 1.55) | 0.78 | 395 | 1.01 (0.67, 1.51) | 0.96 |
| TRANCE | 398 | 0.96 (0.67, 1.40) | 0.85 | 395 | 1.00 (0.68, 1.48) | 1.00 |
| TWEAK | 398 | 0.86 (0.43, 1.70) | 0.66 | 395 | 0.99 (0.48, 2.03) | 0.98 |
|  | Boys |  |  |  |  |  |
|  | *n* | Odds ratio (95% CI) | *p*-value | *n* | Odds ratio (95 % CI) | *p*-value |
| CRP | 429 | 0.97 (0.89, 1.07) | 0.58 | 418 | 0.95 (0.85, 1.06) | 0.38 |
| IL-6 | 445 | 0.97 (0.63, 1.49) | 0.88 | 436 | 0.94 (0.57, 1.56) | 0.82 |
| TGF-α | 445 | 1.42 (0.92, 2.20) | 0.12 | 436 | 1.32 (0.84, 2.09) | 0.23 |
| TRANCE | 445 | 0.85 (0.52, 1.39) | 0.51 | 436 | 0.85 (0.52, 1.40) | 0.53 |
| TWEAK | 445 | 0.46 (0.19, 1.12) | 0.09 | 436 | 0.46 (0.18, 1.15) | 0.10 |

For girls, the adjusted CRP model included the following covariates: smoking, physical activity and chronic disease.

The adjusted models for IL-6, TGF-α, TRANCE and TWEAK included the following covariates: smoking, physical activity and sleep.

For boys, all adjusted models included the following covariates: smoking, physical activity, sleep and chronic disease
