# Supplementary material for: Are pro-inflammatory markers associated with psychological distress in a cross-sectional study of healthy adolescents 15–17 years of age? The Fit Futures study
Source: BMC Psychol. 2022 Mar 15;10:65. doi: 10.1186/s40359-022-00779-8 (PMC8925220; doi:10.1186/s40359-022-00779-8)
Supplement: Supplementary file 4 — Additional file 4. Crude and adjusted associations quartiles of inflammatory proteins and HSCL-10, by linear regressions. [file 40359_2022_779_MOESM4_ESM.docx]

**Additional file 4:** *Crude and adjusted* *associations quartiles of inflammatory proteins and HSCL-10, by linear regressions.*

| Crude analysis | | | | | | Adjusted analysis | | | | |
| --- | --- | --- | --- | --- | --- | --- | --- | --- | --- | --- |
| Girls | | | | | | | | | | |
|  | | | 95 % CI | |  | 95 % CI | | | |  |
| Inflammatory proteins | *n* | B | Lower | Upper | *p*-value | *n* | B | Lower | Upper | *p*-value |
| CRP quartiles | 394 | 0.04 | -0.01 | 0.09 | 0.14 | 393 | 0.02 | -0.03 | 0.06 | 0.51 |
| IL-6 quartiles | 398 | 0.03 | -0.02 | 0.08 | 0.26 | 397 | 0.01 | -0.04 | 0.06 | 0.69 |
| TGF-α quartiles | 398 | <-0.01 | -0.05 | 0.05 | 0.88 | 397 | <-0.01 | -0.05 | 0.05 | 0.95 |
| TRANCE quartiles  (TNF) | 398 | <-0.01 | -0.06 | 0.04 | 0.76 | 397 | <0.01 | -0.05 | 0.05 | 0.90 |
| TWEAK quartiles (TNF) | 398 | -0.02 | -0.07 | 0.03 | 0.51 | 397 | 0.01 | -0.04 | 0.05 | 0.84 |
| Boys | | | | | | | | | | |
|  |  |  | 95 % CI | |  |  |  | 95 % CI | |  |
|  | *n* | *B* | Lower | Upper | *p*-value | *n* | *B* | Lower | Upper | *p*-value |
| CRP quartiles | 429 | <0.01 | -0.03 | 0.03 | 0.99 | 420 | 0.02 | -0.04 | 0.03 | 0.70 |
| IL-6 quartiles | 444 | <0.01 | -0.03 | 0.04 | 0.90 | 435 | -0.01 | -0.04 | 0.03 | 0.65 |
| TGF-α quartiles | 444 | 0.02 | -0.01 | 0.06 | 0.16 | 435 | 0.02 | -0.02 | 0.05 | 0.32 |
| TRANCE quartiles  (TNF) | 445 | -0.03 | -0.06 | 0.01 | 0.15 | 436 | -0.02 | -0.06 | 0.01 | 0.19 |
| TWEAK quartiles (TNF) | 445 | <-0.01 | -0.04 | 0.03 | 0.80 | 436 | <-0.01 | -0.04 | 0.03 | 0.94 |

For girls, the adjusted models for CRP, IL-6, TGF-α and TRANCE included the following covariates: smoking, physical activity and chronic disease.

The adjusted TWEAK model included the following covariates: smoking, snuffing tobacco, physical activity and chronic disease

For boys, adjusted model for all inflammatory markers included the following covariates: physical activity, sleep and chronic disease
